# Custom foot orthoses for chronic metatarsalgia: Study protocol for a participant- and assessor-blinded superiority randomized controlled trial

**DOI:** 10.1371/journal.pone.0340905

**Published:** 2026-01-16

**Authors:** Eléna Payen Schalkens, Maxime Acien, Andrée-Anne Marchand, Pier-Luc Isabelle, Jacques Abboud, Gabriel Moisan

**Affiliations:** 1 Department of Anatomy, Université du Québec à Trois-Rivières, Trois-Rivières, Canada; 2 Groupe de Recherche sur les Affections Neuromusculosquelettiques, Université du Québec à Trois-Rivières, Trois-Rivières, Canada; 3 Departement of Chiropractic, Université du Québec à Trois-Rivières, Trois-Rivières, Canada; 4 Department of Human Kinetics, Université du Québec à Trois-Rivières, Trois-Rivières, Canada; Shahrood University of Technology, IRAN, ISLAMIC REPUBLIC OF

## Abstract

**Background:**

Chronic metatarsalgia (CM) causes significant pain and disability, affecting quality of life. Foot orthoses (FOs) including medially wedged designs with a metatarsal pad decrease excessive plantar pressure under the metatarsal heads, which is a suggested risk factor for developing CM. This FOs model may be effective in diminishing pain and improving function in these individuals. Thus, the objective of this trial will be to compare the effects of medially wedged FOs with a metatarsal pad and sham FOs on pain and foot function in individuals with CM.

**Methods/design:**

This participant- and assessor-blinded superiority randomized controlled trial (RCT) with two parallel groups will be conducted in Trois-Rivières, Canada. Sixty-four participants with CM will be recruited from the Université du Québec à Trois-Rivières outpatient podiatry clinic and via social media invitations. They will be randomized into intervention (customized FOs) or control (sham FOs) groups and will be evaluated at baseline and after 6 and 12 weeks. The primary outcome will be: (1) mean pain during walking for the most painful foot during the past week. The secondary outcomes will be: (1) Foot Function Index, (2) Global rating of change and (3) the 5-level EQ-5D.

**Discussion:**

Medially wedged FOs with a metatarsal pad are expected to provide a greater reduction in pain and improvement in foot function compared to sham FOs. This trial will help guide FOs prescription recommendations for managing foot pain in individuals with CM in the future.

**Trial registration:**

ClinicalTrials.gov NCT06962475

## 1. Background

Foot musculoskeletal disorders cause significant impairments and disabilities for those affected [1]. Among the various types of foot pain, chronic metatarsalgia (CM) is the most prevalent [2]. Metatarsalgia represents 88% of all causes of foot pain [3], with a prevalence of 13–36% in adults [4]. It is characterized by persistent pain to one or more metatarsophalangeal joints, resulting from harm (whether of mechanical origin or not) to the anatomical structures associated with the joint, including bone, cartilage, capsule and ligaments, tendons, bursae and subcutaneous tissue, and skin [3]. Chronic metatarsalgia significantly reduces the quality of life of those affected, physically, psychologically, and socially [5]. Different treatment modalities are used for CM, such as stretching exercises, footwear modifications, and foot orthoses (FOs) [6,7]. The first-line treatment is conservative, and surgery should be considered only when conservative treatment fails. Nevertheless, there is little high-level evidence (level I) to support the efficacy of conservative treatments for CM [8].

The reduction of the mechanical overload under the metatarsal heads during locomotion is a fundamental aspect of CM treatment [7], and it strongly correlates with pain reduction [9]. Foot orthoses are commonly used devices to reduce pain and improve function in individuals with musculoskeletal disorders [10–12]. They reduce forefoot plantar pressure during locomotion in individuals with CM and redistribute plantar pressure more evenly [13,14]. Metatarsal pads, a common FOs modification, effectively redistribute forefoot plantar pressure and thus reduce pain caused by excessive metatarsal loads [9,15–17]. Adding a medial wedge to FOs (inclination in the frontal plane) further reduces forefoot peak pressure in healthy adults [18]. While FOs tested so far are generally effective in managing CM, their effects can be inconsistent across patients, some patients did not find them effective [19–21]. From a biomechanical standpoint, FOs reduce mechanical overload; however, it is crucial to determine whether they also effectively reduce pain and improve foot function. Randomized controlled trials (RCT) are considered the gold standard for evaluating treatment effectiveness and efficiently translating research findings into clinical practice. This study design will allow us to assess whether, in addition to their biomechanical effects, FOs provide clinical benefits for individuals with CM.

The objective of this RCT will be to evaluate the effectiveness of medially wedged FOs with a metatarsal pad in reducing pain and improving foot function in individuals with CM. The hypotheses of this RCT will be that medially wedged FOs with a metatarsal pad will reduce pain and improve foot function compared to sham FOs.

## 2. Materials and methods

### 2.1 Study design

**Fig 1:**
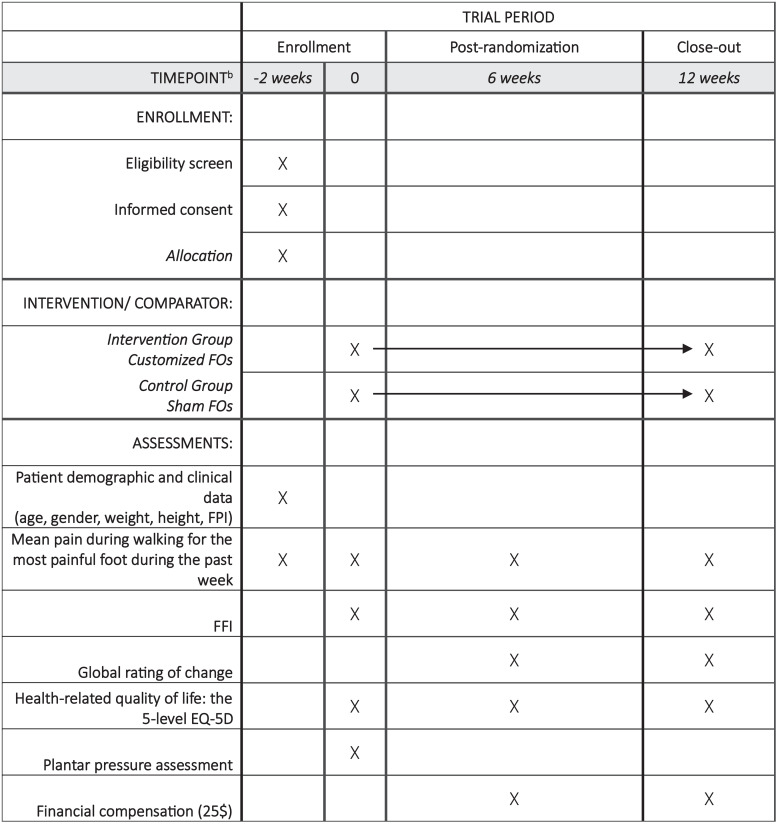
Study schedule of enrollment, interventions, and assessments according to SPIRIT 2013 requirements. (FOs: Foot orthoses; FPI: Foot Posture Index; FFI: Foot Function Index).

The study is a RCT with a parallel-group design, conducted as a participant- and assessor-blinded superiority trial. In this trial, participants with CM will be allocated to either the intervention group (customized FOs) or the control group (sham FOs) (Fig 1). All proposed methods are in accordance with the CONSORT statement which provides relevant guidelines and regulations for RCTs (S1 Checklist) [22]. The study protocol is reported using the Standard Protocol Items: Recommendations for Interventional Trials (SPIRIT) guidelines (Fig 1) (S2 Checklist) [23] and registered on ClinicalTrial.gov (Protocol ID: 2025_UQTR_FOs_RCT; NCT Number: NCT06962475).

### 2.2 Recruitment

#### 2.2.1 Recruitment parameters.

Participants will be recruited from the *Université du Québec à Trois-Rivières* (UQTR) outpatient podiatry clinic and via social media invitations. The estimated start date for recruitment is early January 2026, and the estimated final date is early January 2027. Analysis of preliminary results will be conducted 6 months after the start of recruitment, if half of the participants have been recruited, or as soon as recruitment is completed. Results will be given no later than August 2027, if recruitment has been completed. We will obtain written consent once the participant has been included in the study (validation of inclusion and exclusion criteria and explanation of the protocol). They will be randomized to one of two groups, each receiving a different model of FOs (customized or sham). The total duration of participation will be 4–5 months (from the first contact until the last data collection). Participants will receive $25 after each follow-up (6 weeks and 12 weeks), for a total amount of $50. This financial compensation is intended to compensate participants for their time.

#### 2.2.2 Eligibility criteria.

Participants will be included if they [1] are aged 18 years or over, [2] have bilateral or unilateral metatarsalgia under one or more of the lesser metatarsal heads for at least 3 months (pain score of ≥ 4 out of 10 on a Visual Analogue Scale (VAS)) [24] that is aggravated by weight-bearing activities, [3] are able to walk without assistive devices (e.g., cane, walker), [4] are willing to wear shoes that will accommodate their FOs on a daily basis and [5] are willing to minimize the use of other interventions (e.g., pain medications, physical therapy) during the trial period. If they have bilateral CM, the most painful foot will be used for data collection. The pain score refers to the highest daily pain experienced over the week prior to their enrollment in the study. The CM diagnosis will be made by a podiatrist who, following the definitions of primary and secondary metatarsalgia and excluding other possible pathologies, will determine whether the patient have metatarsalgia (for example: primary: congenital deformities of the metatarsal heads; secondary: synovitis, predislocation syndrome) [7]. Potential participants will be excluded from the study if they have arthritis, neurological diseases (e.g., intermetatarsal neuroma) or other mechanical pain, plantar corns (e.g., intractable plantar keratoma), a history of orthopedic foot surgery, prior use of customized FOs, cognitive impairments, or if they are pregnant.

### 2.3 Interventions

A podiatrist with more than 11 years of clinical experience will take the negative foot impressions using a semi-weightbearing method with a foam box for both groups to ensure adequate blinding of participants and will have them sign the consent form. Then, the foam boxes of the experimental group will be scanned so that customized FOs can be 3D printed, and sham FOs moulded on a plaster replica of the foot. Both groups will receive identical clinical guideline-based information and support at the beginning of the trial. The instructions will be as follows: [1] undertake a familiarization protocol of 5 to 14 consecutive full days [25], [2] wear the FOs (customized or sham) at all time when they are standing (targeted minimum of 5 hours per day), [3] avoid positions that cause the metatarsophalangeal joints to dorsiflex (e.g., squatting or tiptoeing), [4] mechanical stress load management according to the tissue stress model [26], [5] not to walk barefoot and [6] wear shoes with good cushioning in which the FOs will be properly placed.

#### 2.3.1 Customized foot orthoses.

**Fig 2:**
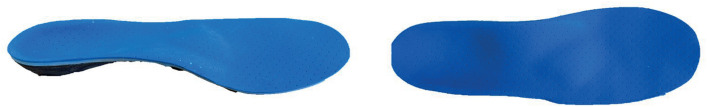
Customized foot orthoses (left: medial view, right: top view).

Participants randomized to the intervention group will receive customized FOs for both feet. The customized foot orthoses will be manufactured with a Nylon (PA11) shell with 6° medially wedged forefoot-rearfoot posts (2.6 mm if the participants’ weight (PW) is inferior or equal to 45 kg; 3.2 mm if 45 < PW ≤ 100 kg; 4.0 mm if PW > 100 kg), a full-length 3 mm Poron top cover, a metatarsal pad (15 shore A) located 5 mm proximal to the metatarsal heads [9], a 1.5 mm Multiform top cover on top of a 1 mm Black Leatherette layer (Fig 2).

#### 2.3.2 Sham foot orthoses.

**Fig 3:**
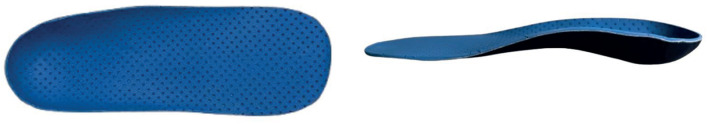
Sham foot orthoses (right: medial view, left: top view).

Participants randomized to the control group will receive sham FOs for both feet. The molded sham FOs will be manufactured from 3 mm ethylene vinyl acetate on top of a 1 mm Black Leatherette layer. They will have an identical top cover material, color and a similar shape than the customized FOs, however, they will provide negligible mechanical support, considering the very low stiffness of their medial arch. The only impact of these FOs on plantar pressure is under the heel [27]. These devices have been used as a sham condition in previous trials [28] and a study has validated them as being credible [27] (Fig 3).

### 2.4 Randomization

All included participants will be randomized into one of the two groups (intervention or control) using a minimization method with an 80:20 probabilistic element to reduce predictability (allocation ratio of 1:1, deterministic minimization) to ensure that both groups will be balanced with respect to predetermined criteria. These criteria have been identified based on factors known susceptible to influence the response to the treatment: gender, age and mean pain during walking for the most painful foot during the past week (first visit) [29]. The findings from a recent study showed that metatarsalgia is most common in middle aged women (60%) and with an average age of 54 years [30]. Participants will be added one by one; each profile will be studied by the code so that its entry into the group limits the differences between the groups as much as possible, a member of research team (MA) will place the group in which the participant is assigned in a sealed envelope. Each envelope will be opened at the end of the baseline assessment by a certified orthotist to manufacture the appropriate model of FOs according to the allocated group.

### 2.5 Data collection

Once the FOs are manufactured, participants will return to UQTR to collect them. During this visit, they will complete the initial questionnaires, and plantar pressure measurements will be taken with and without FOs. The plantar pressure will be collected with a Pedar-X in-shoe pressure measurement system (Novel Corporation, Munich, Germany), sampling at 100 Hz. The insoles of this system will be inserted between the foot and either the insole of the shoe or the FOs. Once they are equipped with the plantar pressure system, the participants will be allowed five minutes of acclimatization. Then, they will walk 10 times at a self-selected speed on a 10-meter walkway. The second and third assessments will be completed by phone or visioconference by a member of the research team, blinded to the group allocation of the participants. We will use MATLAB R2024b (The MathWorks Inc., Boston, MA, USA) for randomization and Qualtrics for the questionnaires.

### 2.6 Blinding and monitoring

Participants will be blinded as to which group they are allocated to and will not see the other participants. The researchers performing data collection as well as those performing statistical analyses will also be blinded to the group allocation. Participants will be advised that the trial has been designed to compare one of two types of orthopedic insoles for CM, but they will not be informed about the specific characteristics of the interventions. Given the nature of the intervention (i.e., FOs), it is not possible to blind the research staff responsible for their administration. To maintain consistency, the interventions will be described as “orthopedic insoles” throughout the study. If their FFI increases by 20% or more, then they will be prevented from continuing the study to avoid worsening the symptoms. The participants will complete a daily log of foot orthoses wear time and the use of various interventions (with text messages), if they do not wear their foot orthoses for a minimum of 3 hours per day on average, they will be excluded from the study. All adverse events will be recorded, assessed for severity and relatedness to the intervention, and reported to the Trial Sterring Committee.

### 2.7 Participant trajectories

Regardless of group allocation, all participants will continue to follow their usual care and minimize the use of other interventions (e.g., pain medications, physical therapy) during the study period. All medication will be monitored throughout the trial.

### 2.8 Outcomes

#### 2.8.1 Primary outcomes.

- Mean pain during walking for the most painful foot during the past week: visual analog scale (from 0 to 10, 0 being no pain and 10 pain as bad as it could be).

#### 2.8.2 Secondary outcomes.

- Foot Function Index (FFI): The FFI is a widely used, valid, and reliable self-administered questionnaire consisting of 23 items grouped into 3 domains: foot pain (9 items), disability (9 items), and activity limitation (5 items) [31].
- Global rating of change (GROC): participants’ perception of overall treatment effect will be measured using the self-reported global rating of change scale [32]. This outcome will then be dichotomized into the categories of “effective” (“a very great deal better”, “a great deal better”, “a good deal better” and “moderately better”) and “ineffective” (“somewhat better”, “a little better”, “about the same, hardly any better at all”, “no change”, “about the same, hardly any worse at all”, “a little worse”, “somewhat worse”, “moderately worse”, “a good deal worse”, “a great deal worse” and ‘a very great deal worse”) [33].
- Health-related quality of life (HRQoL): the 5-level EQ-5D (EQ-5D-5L) will be used [34]. The descriptive system comprises five dimensions: mobility, self-care, usual activities, pain/discomfort and anxiety/depression. Each dimension has 5 levels: no problems, slight problems, moderate problems, severe problems and extreme problems. The participant will be asked to indicate his/her health state by ticking the box next to the most appropriate statement in each of the five dimensions. This decision results in a 1-digit number that expresses the level selected for that dimension. The digits for the five dimensions can be combined into a 5-digit number that describes the patient’s health state.

#### 2.8.3 Other variables.

- Gender: Male, female or other.
- Age: in years (from the date of birth).
- Weight: in kg (measured in clinic).
- Height: in meters (measured in clinic).
- Body Mass Index (BMI): will be calculated with the formula weight (Kg) divided by height squared (meters^2^).
- Foot Posture Index (FPI): The six clinical criteria used in FPI are: 1. Palpation of the talus head (medial and lateral deviation of the talus head in relation to the navicular bone); 2. Curvature of the supra and lateral inframaleolar region; 3. Position of the calcaneus in the frontal plane; 4. Prominence of the talonavicular region; 5. Congruence of the internal longitudinal arche; and 6. Abduction/adduction of the forefoot with respect to the rearfoot. Each criterion is rated between −2 and +2 and the sum of all gives a total score indicating the foot posture (Normal = 0 to +5; pronated = +6 to +9; highly pronated = +10 to +12; supinated = −1 to −4 and highly supinated = −5 to −12).
- Effects of the FOs on plantar pressure: peak plantar pressure during a stance phase (kPa) under the metatarsal heads, measured with a Pedar-X in-shoe pressure measurement system (Novel Corporation, Munich, Germany). This system is repeatable and can be used as a valuable tool in the assessment of in-shoe plantar pressure distribution [35–37].

### 2.9 Sample size

To calculate pooled variance (σp²) and square of detectable difference (Δ²), we relied on pain data from a 3-month clinical trial with customized foot orthoses [6] and sham FOs [38].

- **Pooled standard deviation:** σ_p_ ≈ 2.71 → σ_p_² ≈ 7.34
- **Detectable difference:** Δ = 1.49 → Δ² ≈ 2.22
- **Justification of Δ:** δ = 1.49 corresponds to the minimum clinically important difference on the VAS (≈15% of the scale), based on the study by Reina-Bueno, Vázquez-Bautista [38].
- α = 0.05
- β = 0.2
- **Sum of critical values squared:** (Z_1−α/2_ + Z_1−β_)² = (1.96 + 0.84)² ≈ 7.84

Considering the longitudinal design with three repeated measurements per participant (baseline, 6 weeks and 12 weeks), we estimated the effective variance per subject using a linear mixed-effects model with random intercept:


$${var}_{subj}=\;\sigma_{b}^{\text{2}}+\frac{\sigma_{w}^{\text{2}}}{m}\approx \text{1}.\text{4}\text{6}\text{8}+\;\frac{\text{5}.\text{8}\text{7}\text{2}}{\text{3}}\approx \text{3}.\text{4}\text{2}\text{5}$$

Using this variance, the sample size formula for two independent groups:


$$n\approx \frac{\text{2}\times ({\text{Z}}_{\text{1}-\alpha /\text{2}\;}+\;{\text{Z}}_{\text{1}-\beta })²\;\times {var}_{subj}}{\Delta^{\text{2}}}\approx \text{2}\text{5}$$

Therefore, the minimal sample size would be 25 per group. Considering an attrition rate of 20%, the final sample size should be 32 participants per group, resulting in a total sample size of 64 participants to detect significant differences for the primary outcome (VAS pain) at the primary timepoint (12 weeks). A sensitive analysis table showing required sample sizes under the different correlations (ρ = 0.3, 0.5, 0.7) is available in Supplementary Table S3 in S1 File.

### 2.10 Statistical analyses

All analyses will follow the intention-to-treat principle, with additional per-protocol sensitivity analyses. Baseline characteristics will be summarized using means for continuous variables and frequencies (%) for categorical variables, without statistical testing between groups.

For the primary outcome (VAS pain), between-group differences will be analyzed using linear mixed-effects models, with participants included as random effects and baseline scores and minimization factors included as fixed covariates. The primary analysis will focus on the change from baseline to week 12, adjusted for baseline values, to provide a more precise estimate of the treatment effect. This approach allows all available baseline and follow-up data to be used, even in the presence of missing outcomes, under the assumption that data are missing at random. If the assumption of normally distributed residuals is violated, generalized linear mixed models will be considered as an alternative.

Secondary continuous outcomes (FFI – foot pain, FFI – disability, FFI – activity limitation, EQ-5D-5L index) will be analyzed using the same approach. The Global Rating of Change (GROC) will be modelled using ordinal logistic regression; a dichotomized responder analysis (effective vs ineffective) will be presented as exploratory. No formal multiplicity adjustments will be applied, but results will be interpreted with caution.

If missing data exceed 10% for the primary outcome, multiple imputation by chained equations will be used as a sensitivity approach, including all variables related to missingness and outcome prediction in the imputation model. Sensitivity analyses will also be performed under non-Missing At Random assumptions (e.g., using delta-adjustment or pattern-mixture models) to assess the robustness of the findings to departures from the Missing At Random assumption.

Concomitant care and rescue medication use will be recorded descriptively throughout the trial and summarized by treatment group. If substantial between-group differences are observed, these variables will be considered in sensitivity analyses.

The plantar pressure will be divided in nine-foot regions [39]. The analyses will be analyzed with Statistical Parametric Mapping (SPM1D, www.spm1d.org). We will assess the normality of the distribution of the peak plantar pressure (spm1d.stats.normality.anova1rm function) and followed by SPM paired t-tests SPM(t) as post hoc analyses will be used to compare the peak plantar pressure across with and without FOs, when the data will be normally distributed. The non-parametric version, SnPM(t) tests, will be use when data will not normally distributed.

All this will be carried out in frequency distribution tables of different categories, using SPSS (IBM SPSS Statistics: V.28, USA) and plantar pressure will be implemented in MATLAB R2024b (The MathWorks Inc., Boston, MA, USA) using open access scripts (www.spm1d.org).

No additional analyses (e.g., subgroup and adjusted analyses) are planned for this study. The two-sided level of p will be set at 0.050.

### 2.11 Data management

The researchers will fill in data to the data collection sheet accurately, completely, and timely based on original observations. EPS will be responsible to fill in data collection sheets during each interview. All data collected during this study are totally confidential and will never allow participant identification. Confidentiality will be assured by replacing the participants’ names by an alphanumeric code. EPS will be blind after the recruitment of participants and will know the allocation after the last interview. The data collected will be stored under lock and key in the evaluation or intervention room (paper documents) or on a secure network, with both network and electronic document access protected by passwords under the responsibility of the Groupe de Recherche sur les Affections Neuromusculosquelettiques at the Université du Québec à Trois-Rivières. Only team members will have access to these data. All of them have signed a confidentiality agreement. For randomization purposes, MA will have access to baseline data.

All data of the randomized controlled trial will be anonymized and filed on Borealis 5 years after the end of the study. Data will be available upon reasonable request, in accordance with participant consent. Any data essential to replicate the results are included in the manuscript and/or supporting information files.

### 2.12 Composition of the coordinating center and trial steering committee and plans for auditing trial conduct

The coordinator of the study will be EPS. She will be responsible for all aspects of the local organization including identifying potential participants and obtaining consent from participants. PLI (certified podiatrist) is the clinician who will check the inclusion and exclusion criteria, make the diagnosis, make the foot impressions and give the information on the use of the FOs. MA is a doctoral student and will collect the plantar pressure data during the first assessment and is responsible for randomizing the participants. This study is supervised by JA and GM. Both are professors at UQTR and are supervising EPS’s doctoral studies. The local Trial Steering Committee (TSC) is composed of EPS, AAM, JA and GM. The team discussed the test protocols, the intervention content, and the related materials together. The committee will meet bimonthly throughout the study. If any modifications to the protocol need to be addressed or if any adverse events occur, the TSC will discuss the situation as soon as possible and will inform the research ethics committee. Independent Data Monitoring Committee is not planned for this single-center, low-risk trial. The monitoring will be conducted by the principal investigator of the study each month as an audit of trial conduct. There is no stakeholder and public involvement group for this study. Only the EPS, AM, IPL, AAM, JA and GM will have authorship eligibility.

### 2.13 Ethics and dissemination

This study will be conducted in accordance with the Helsinki Declaration [40]. This study does not involve the collection of biological specimens for storage. The results will be communicated to participants, healthcare professionals, the public, and other relevant groups via publications in scientific journals, platform presentations, and poster presentations during national and international conferences.

### 2.14 Abbreviations

CM: Chronic Metatarsalgia
FOs: Foot Orthoses
RCT: Randomized Controlled Trial
FPI: Foot Posture Index
FFI: Foot Function Index
VAS: Visual Analogue Scale
GROC: Global Rating of Change
HRQoL: Health-related quality of life
BMI: Body Mass Index
SPM1D: Statistical Parametric Mapping-1D
TSC: Trial Steering Committee

### 2.15 Declaration

**Ethics approval and consent to participate:** The proposed study protocol was approved by the UQTR Human Research Ethics Board (CER-25-319-07.03). Written informed consent will be obtained from all participants before completing the baseline data collection.

## 3. Discussion

The customized FOs selected in this RCT are among the best method currently available to reduce plantar pressure under the forefoot. Monitoring over several weeks with intermediate results will allow us to see if there is a time when the effects of the treatment stagnate or become more pronounced. It is anticipated that the study will provide valuable evidence for improvement of the treatment of CM.

### 3.1 Limitations

The sham FOs that we will use are a credible intervention (i.e., participants rated them as credible) and have the same effects on plantar pressure under the midfoot and forefoot than shoes [27]. However, biomechanical analyses of these orthoses showed that they reduce plantar pressure under the heel compared to shoes [27]. By evaluating the effects of the experimental conditions (Customized and sham FOs) during the baseline sessions, we will ensure that customized FOs reduce plantar pressure under the lesser metatarsal heads and that sham FOs provide negligible effects.

Although participants will be asked to limit the use of anti-inflammatory drugs and medications, it will not be possible to prevent them from taking these. This intake may impact the pain measured in the primary outcome.

### 3.2 Strengths

This study will be a RCT, the gold standard to determine interventions effectiveness, so the bias possible will be reduced. By reducing bias, the possibility that the examination of the causal relationship between FOs and outcomes is distorted by external elements will be limited. This research will have several strengths, such as the random assignment to the treatment and the blinding of the evaluators, the direct applicability of the results obtained because the prescriptions and instructions given are like those given in the clinic. This trial will help to guide FOs prescription recommendations for the management of foot pain for people with CM in future.

## Supporting information

S1 Checklist CONSORT Checklist: Checklist of items to include when reporting a randomized trial, according to the CONSORT 2010 guidelines. (DOCX)

S2 Checklist SPIRIT Checklist: Checklist of recommended items to address in a clinical trial protocol, according to the SPIRIT 2013 guidelines. (DOCX)

S1 File Sensitivity analysis of sample size for different within-subject correlations. (DOCX)
