# Supplementary material for: Custom foot orthoses for chronic metatarsalgia: Study protocol for a participant- and assessor-blinded superiority randomized controlled trial
Source: PLoS One. 2026 Jan 16;21(1):e0340905. doi: 10.1371/journal.pone.0340905 (PMC12810774; doi:10.1371/journal.pone.0340905)
Supplement: S1 File — (DOCX) [file pone.0340905.s003.docx]

| **Within-subject correlation (rho)** | **Effective variance per subject** | **Sample size per group** | **Sample size per group (with 20% attrition)** |
| --- | --- | --- | --- |
| 0.3 | ≈3.79 | 27 | 34 |
| 0.5 | ≈3.43 | 25 | 32 |
| 0.7 | ≈3.07 | 23 | 29 |

**Supplementary Table S3 – Sensitivity analysis of sample size for different within-subject correlations**
